# Comprehensive Membrane *N*-Glycoproteomics Using Human Breast Cancer Cell Line Pairs

**DOI:** 10.5702/massspectrometry.A0117

**Published:** 2023-04-11

**Authors:** Daisuke Takakura, Haruka Yoshida, Shoko Ohashi, Nana Kawasaki

**Affiliations:** 1 Graduate School of Medical Life Science, Yokohama City University, 1–7–29 Suehiro-cho, Tsurumi-ku, Yokohama 230–0045, Japan

**Keywords:** membrane glycoproteins, glycosylation, *N*-glycoproteomics, LC/MS/MS

## Abstract

Aberrant glycosylation of membrane proteins is a hallmark of cancer and a useful molecular marker for the diagnosis of breast cancer (BC). However, the molecular mechanisms by which altered glycosylation affects the malignant transformations associated with BC are poorly understood. Accordingly, we performed comparative membrane *N*-glycoproteomics using the human BC cell line pair, Hs578T, and its syngeneic normal cell line, Hs578Bst. A total of 359 *N*-glycoforms derived from 113 proteins were identified in both cell lines, of which 27 were found only in Hs578T cells. Significant changes in *N*-glycosylation were found in the lysosome-associated membrane protein 1 (LAMP1), the integrin family, and laminin. Confocal immunofluorescence microscopy images revealed the accumulation of lysosomes in the perinuclear space in cancer cells, which could be associated with marked changes in LAMP1 glycosylation, such as a decreased level of polylactosamine chains. Overall, the alterations in glycosylation may be involved in changes in the adhesion and degradation of BC cells.

## INTRODUCTION

The prevalence of breast cancer (BC) has increased over the last few years. In fact, BC is now one of the most common malignancies that affect women worldwide.^1)^ BC is classified into heterogeneous subtypes, leading to diverse pathological manifestations associated with complex molecular mechanisms.^2)^ No single therapeutic approach is universally beneficial for BC owing to its diverse clinical course. As a result, various omics approaches, including genomics,^3,4)^ transcriptomics,^5)^ and proteomics,^6)^ have been utilized so as to better understand the molecular nature of BC.

Protein glycosylation is a major post-translational modification in mammalian cells. Nearly all plasma membrane proteins are glycosylated,^7)^ and their carbohydrate moieties play important roles in various biological events, such as adhesion, cell recognition, cell–cell interactions, and signal transduction.^8–10)^ Indeed, there is increasing evidence to show that glycosylation has an important role in cancer cell proliferation, invasion, and metastasis.^11,12)^ Aberrant core fucosylation of the cell surface receptor CD276 antigen is a profound immunosuppressive factor in BC.^13)^ High mannose and complex-type *N*-glycans, which increase BC progression, are involved in cell growth signaling, the activation of invasion and metastasis, and tumor-promoting inflammation.^14)^ The dysregulation of mannosyloligosaccharide 1,2-α-mannosidase, an oligosaccharide, is associated with the progression of BC.^15)^ However, the molecular mechanisms by which altered glycosylation affects the malignant transformation of BC are poorly understood.

Glycoproteomics using mass spectrometry (MS) is the most useful method for analyzing glycosylation changes associated with the malignant transformation of cancer. In general, *N*-glycopeptides enriched by hydrophilic interaction chromatography,^16,17)^ lectin affinity,^18–20)^ and acetone^21,22)^ are subjected to liquid chromatography/tandem mass spectrometry (LC/MS/MS) to not only the deduce peptide sequence, but also the glycosylation site and glycan composition. A comprehensive analysis of the changes in glycosylation during cancer progression using MS-based glycoproteomics would be expected to clarify the role of glycans in the malignant transformation of cancer.

Cancer-derived cell pairs matched with normal cells from the same patient are valuable resources for cancer research. In BC, Hs578T and its syngeneic normal cell line, Hs578Bst, are the only cell pairs with the same tissue source of normal and cancer cells and have been well characterized.^23–25)^ Hs578T and Hs578Bst serve as novel and promising model systems for studying the molecular mechanisms of cancer.^26)^ In this study, we conducted comparative *N*-glycoproteomics to determine whether *N*-glycosylation contributes to malignancy using the human BC cell line pairs, Hs578T and Hs578Bst.

## MATERIALS AND METHODS

### Cell culture

Two syngeneic cell lines, normal breast Hs578Bst (HTB-125) and triple-negative breast tumor Hs578T (HTB-126), were obtained from the American Type Culture Collection (ATCC, Manassas, VA, USA). Normal breast cells were cultured in Hybri-Care Medium (ATCC) supplemented with 10% heat-inactivated bovine serum (FBS, Bovogen Biologicals, Melbourne, Australia), 1% penicillin/streptomycin (Gibco Invitrogen; Thermo Fisher Scientific, Waltham, MA, USA), and 30 ng/mL mouse EGF, while the breast cancer cells were cultured in Dulbecco’s Modified Eagle’s Medium (DMEM, Gibco Invitrogen), 1% penicillin/streptomycin, and 0.01 mg/mL human insulin (Gibco Invitrogen) at 37°C in the presence of 5% CO_2_. The cells were washed three times with phosphate-buffered saline (PBS) containing 1% protease inhibitor cocktail (Sigma, St. Louis, MO, USA) at 4°C, dissociated using StemPro Accutase (Gibco Invitrogen), and centrifuged at 900 rpm for 5 min. The biological replicates were cultured under the same conditions.

### Trypsin digestion and glycopeptide enrichment

The acetone-based glycopeptide enrichment method was conducted as described previously.^21)^ Integral membrane-associated proteins were extracted from the two cell lines using the ProteoExtract Native Membrane Protein Extraction Kit (Calbiochem; Merck, Darmstadt, Germany). All membrane proteins were quantified using the bicinchoninic acid method, and 30 μg of the membrane proteins with 1% sodium deoxycholate were reduced with 10 mM dithiothreitol at 60°C for 40 min and alkylated with 20 mM iodoacetamide at 25°C in dark for 30 min. The membrane proteins were subsequently digested with 1.5 μg of modified trypsin (Promega, Madison, WI, USA) for 16 h at 37°C. Glycopeptides were precipitated with a five-fold volume of ice-cold acetone, incubated for 16 h, and then centrifuged at 12,000×g for 10 min. A portion of the precipitate from both cell types was dissolved in 30 μL of 20 mM phosphate buffer (pH 7.2). To release the *N*-glycans, the sample was incubated with 2U PNGase F (Roche, Basel, Switzerland) containing 50% glycerol 16 h at 37°C. The deglycosylated peptides were desalted using a C-18 Spin Columns (Pierce; Thermo Fisher Scientific) and then dried with a SpeedVac concentrator.

### LC/MS/MS and identification of the *N*-glycopeptides

Peptides and glycopeptides were separated on an EASY-nLC 1000 (Thermo Fisher Scientific) using an L-column2 ODS trapping column (0.3×5 mm, 5 μm; Chemicals Evaluation and Research Institute, Tokyo, Japan) and a nano HPLC capillary column (75 μm×120 mm, 3 μm, C18; Nikkyo Technos, Tokyo, Japan). The mobile phase consisted of water containing 0.1% v/v formic acid (Pump A) and acetonitrile containing 0.1% v/v formic acid (Pump B). The sample injection volume was 5 μL. The peptides and glycopeptides were eluted at a flow rate of 0.3 μL/min with a linear gradient from 0 to 45% B over 115 min. Mass spectra were acquired on a Q Exactive mass spectrometer (Thermo Fisher Scientific) equipped with a Nanospray Flex Ion Source (Thermo Fisher Scientific) operated in the positive ion mode. The Xcalibur 4.0 workstation (Thermo Fisher Scientific) was used for MS control and data acquisition. The spray voltage was set at 2.0 kV, the capillary temperature was maintained at 250°C, and the S-lens (stacked ring ion guide) RF (radio frequency) level was 50. Full mass spectra were acquired using an *m*/*z* range of 350–2000 for deglycosylated peptides or 700–2000 with a resolution of 70,000. Product ion mass spectra were acquired against the 10 most intense ions using a data-dependent acquisition method with a resolution of 17,500, normalized collision energy of 27%, and exclusion of 17s.

Raw data files were processed using the SEQUEST search engine (Thermo Fisher Scientific) for peptides and the Byonic search engine ver. 3.11 (Protein Metrics, Cupertino, CA, USA) for glycopeptides, integrated as a node in Proteome Discoverer ver. 2.4 (Thermo Fisher Scientific). The UniProtKB database for humans (status/2021/07) and *N*-glycan database (307 *N*-glycans) were used for searching the database. The search conditions were as follows: trypsin, maximum number of missed cleavages, 2; static modification, carbamidomethylation (C); dynamic modifications, Gln>PyroGlu (N-term Q), oxidation (M), deamidation (N), precursor mass tolerance of 10 ppm, fragment mass tolerance of 20 ppm, and maximum number of *N*-glycosylations per peptide, 2. After the search, all identified peptides were filtered at a false discovery rate (FDR) threshold of 1% using a target/decoy search strategy. The identified peptides were evaluated for reliability using the percolator node. Only high-confidence *N*-glycoforms, glycopeptides with different glycan structures attached to the same peptide backbone, were processed using Microsoft Excel ver. 2209 (Microsoft, Redmond, WA, USA).

### Glycoproteomics data analyses

Label-free quantification of the *N*-glycoforms was performed using a combination of a Minora Feature Detector, Feature Mapper, and Precursor Ions Quantifier nodes in Proteome Discoverer 2.4 (Thermo Fisher Scientific). The analytical conditions were as follows: Peptide to Use, unique; Precursor Quantification, area; and normalization mode, none.

The Gene Ontology (GO) of the de-glycosylated proteins was determined using the Database for Annotation, Visualization, and Integrated Discovery (DAVID) Bioinformatics Resources 6.8 (https://david.ncifcrf.gov/home.jsp). Principal component analysis (PCA) was used to compare the glycoform peak areas between Hs578T (BC) and the normal breast Hs578Bst cell samples. The log2 fold change for *N*-glycoforms were plotted against the −log10 of the *p*-value. Statistical significance was declared at FDR LogWorth (−log10 *p*-value) of 1.3 (equivalent of a *p*-value of 0.05). The presence of signal peptides and transmembrane (TM) segments was predicted using SignalP (ver.5.0, https://services.healthtech.dtu.dk/service.php?SignalP-5.0) and TMHMM Server (ver. 2.0, https://services.healthtech.dtu.dk/service.php?TMHMM-2.0). Statistical analyses were performed using JMP Pro 15 software (SAS Institute, Cary, NC, USA). To predict the interaction pattern of aberrant *N*-glycoproteins in BC cells, the protein–protein interaction (PPI) network was analyzed using the Search Tool for the Retrieval of Interacting Genes/Proteins (STRING) database (https://string-db.org).^27)^

Changes in site-specific glycosylation in aberrant glycoproteins were examined *via* manual spectral analysis. The *N*-glycoforms identified using the Byonic search engine were supported by manual assignments. The typical oxonium ions were annotated on the spectra, including *m*/*z* at 204.08 (HexNAc), 274.09 and 292.10 (NeuAc), 325.11 (Hex-Hex), and 366.14 (HexNAc+Hex). The peptide and glycan masses of the glycopeptides were deduced from the molecular masses of the peptide ion carrying a single *N*-acetylglucosamine, which is commonly found to be more intense. The remaining glycoforms were determined from the mass intervals between the glycoforms, and their peak areas were calculated from the extracted ion chromatogram (XIC) and summed across all charge states of the *N*-glycoforms. The site-specific glycan profiles were compared using mean normalized peak area values. The relative peak area of each glycoform was normalized by the mean of the peak area value for all glycoforms derived from the same glycosylation site to enables comparative analysis of changes in glycan profiles of proteins with different expression levels.^28)^

### Immunoblotting

The integral membrane-associated proteins (1.5 μg per well) were solubilized in EzApply (ATTO, Tokyo, Japan) and denatured for 5 min at 95°C. Following separation by SDS-PAGE using 5–20% precast gel (ATTO, Tokyo, Japan) under a constant current of 20 mA for 80 min, the proteins were transferred onto a PVDF membrane using a Lightning Blotter (PerkinElmer, Waltham, MA, USA). The PVDF membrane were blocked with 5% ECL Blocking Agent (Cytiva, Marlborough, MA, USA) in PBS containing 0.1% Tween 20 (PBS-T) for 1 h. After washing three times with PBS-T, the PVDF membrane was incubated overnight at 4°C with the primary antibodies against lysosome-associated membrane glycoprotein 1 (LAMP1) (1 : 1,000 dilutions). The membrane was washed three times with PBS-T, and incubated with an HPR conjugated anti-rabbit secondary antibody for 1 h. The PVDF membrane was washed three times with PBS-T, the target glycoproteins were detected using ECL Western Blotting Substrate (Pierce, Thermo Fisher Scientific), and visualized by LAS-3000 Imager (Fujifilm, Tokyo, Japan).

### Immunofluorescence confocal microscopy

BC and control cells were cultured in glass-bottomed 12-well plates at 37°C in the presence of 5% CO_2_ for 48 h. Cells on glass coverslips were washed three times with PBS and fixed with methanol (1 mL). After two rounds of washing with PBS, the cells were blocked with 5% albumin in PBS for 16 h at 4°C and then incubated with the primary antibodies in 5% albumin in PBS for 2 h at 37°C. Primary antibodies against LAMP1 (ab25630, Abcam, Cambridge, UK) and cathepsin D (#2284, Cell Signaling Technology, Danvers, MA, USA) were diluted 1 : 20 and 1 : 200, respectively. After three rounds of washing with 0.05% triton in PBS for 5 min, the cells were incubated with the relevant secondary antibody in 5% albumin in PBS for 30 min at 37°C in the dark. The secondary antibodies, fluorescein isothiocyanate (FITC) goat anti-mouse IgG (ab7064, Abcam) and Alexa Fluor 555 donkey anti-rabbit IgG, were diluted 1 : 200. After three, 10 min rounds of washing with 0.05% triton in PBS in the dark, the cells were mounted with ProLong Diamond Antifade Mountant with DAPI (Molecular Probes; Thermo Fisher Scientific). Finally, the cells were visualized using a TCS SP8 confocal microscope (Leica, Wetzlar, Germany).

## RESULTS

### Glycopeptide enrichment and *N*-glycoproteomics

**Figure 1:**
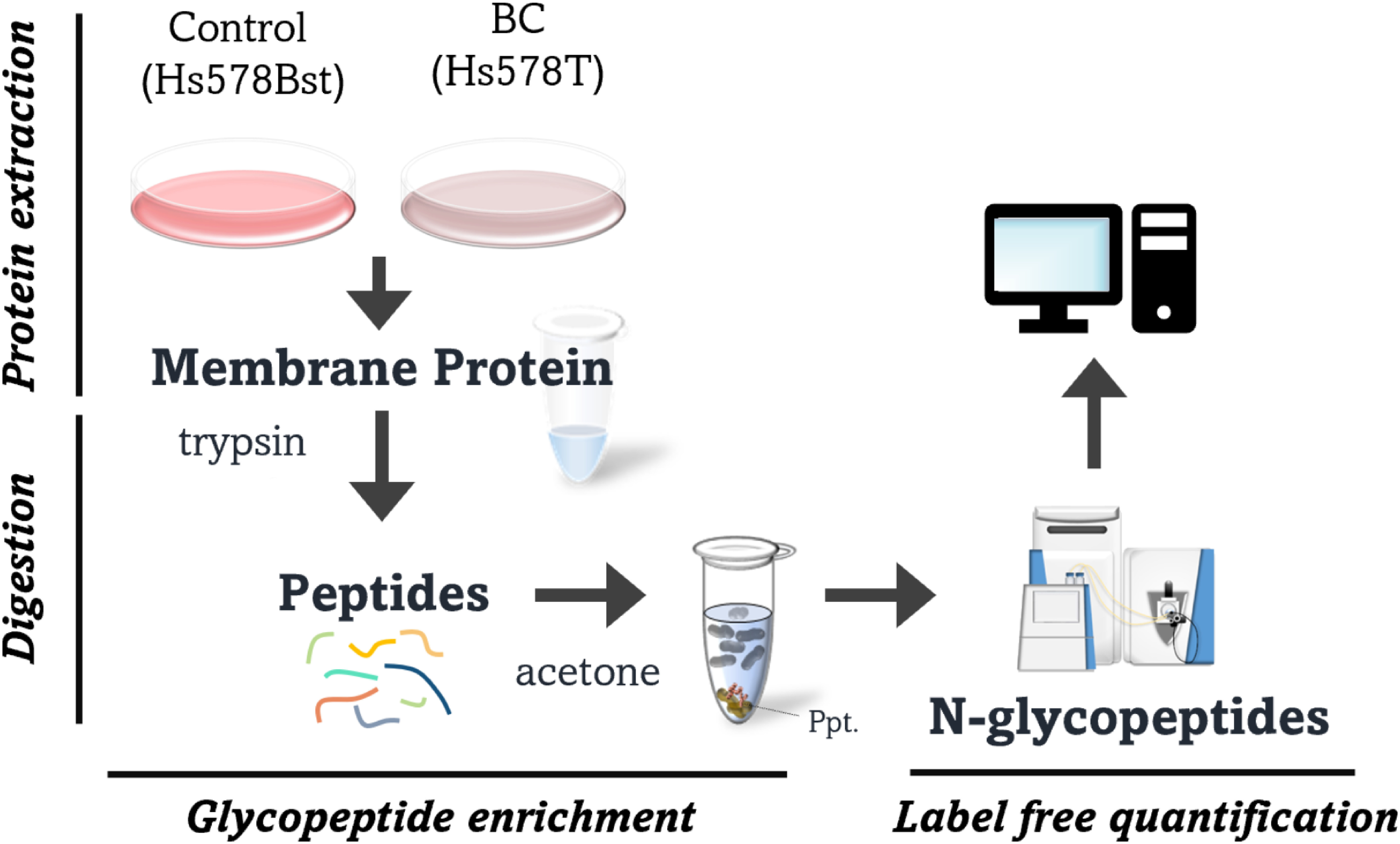
Fig. 1. Schematic workflow of *N*-glycoproteomics.

**Figure 2:**
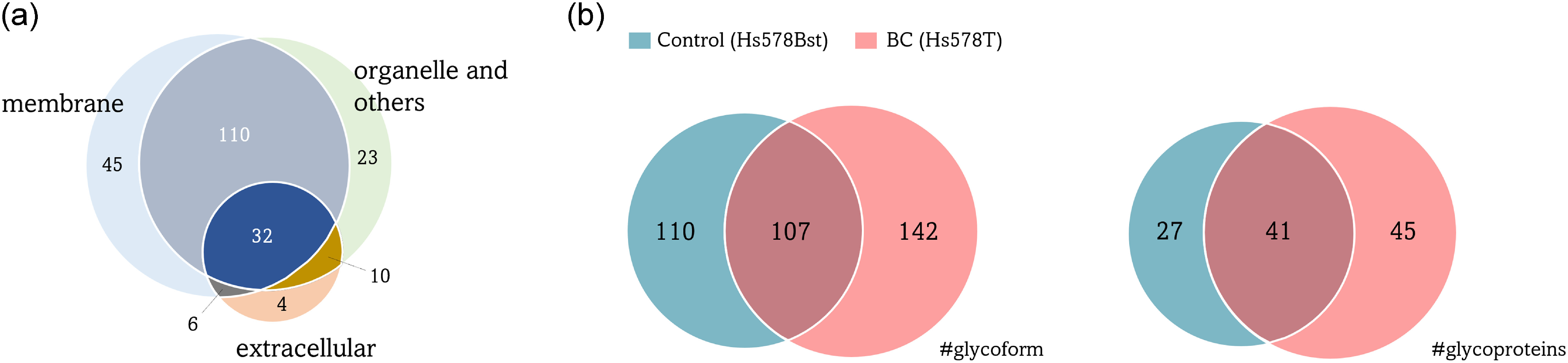
Fig. 2. Qualitative comparison of the *N*-glycopeptides identified by LC/MS/MS.

The strategy used in our glycoproteomic study with the two syngeneic cell lines, Hs578Bst and Hs578T, is summarized in Fig. 1. Membrane-associated proteins, which are the major glycoproteins, were subjected to reductive alkylation followed by tryptic digestion. Thereafter, the glycopeptides were enriched *via* acetone precipitation. A database search for the conversion of Asn to Asp revealed 419 potential glycopeptides from the 230 proteins in both samples. When these glycoproteins were used for Gene Ontology (GO) annotations, approximately 40% (86 proteins) of the identified proteins were classified as “plasma membrane proteins,” whereas the remaining proteins were categorized into extracellular exosome and/or organelle membranes, such as the ER, Golgi, and lysosomes (Fig. 2a). Figure 2b shows a qualitative comparison of the *N*-glycoforms identified using LC-MS/MS. LC-MS/MS revealed 249 and 217 *N*-glycoforms in the BC and control cell samples, respectively. A total of 359 membrane-related *N*-glycoforms from 113 proteins were identified. Among these, 142 *N*-glycoforms were found in BC cells, 110 were found in the control, and 107 were found in both samples. The MS raw data files were deposited in the ProteomeXchange Consortium *via* the jPOST partner repository (http://jpostdb.org) with the dataset identifiers PXD040130.

The comprehensive analysis of intact *N*-glycopeptides was divided into four stages. Step 1: Preparation of membrane proteins; Step 2: Trypsin digestion; Step 3: Concentration of *N*-glycopeptides using acetone; Step 4: Label-free quantification of *N*-glycoforms.

(a) Subcellular localization of the *N*-glycoproteins. Most glycoproteins were found in the membrane (84%), including the plasma membrane, cell surface, and basement membrane, followed by organelles (76%), including ER, Golgi, and lysosomes, and extracellular regions (23%). (b) Venn diagram showing the distribution of the *N*-glycoforms identified in BC and control cell samples.

### BC-related *N*-glycoforms

**Figure 3:**
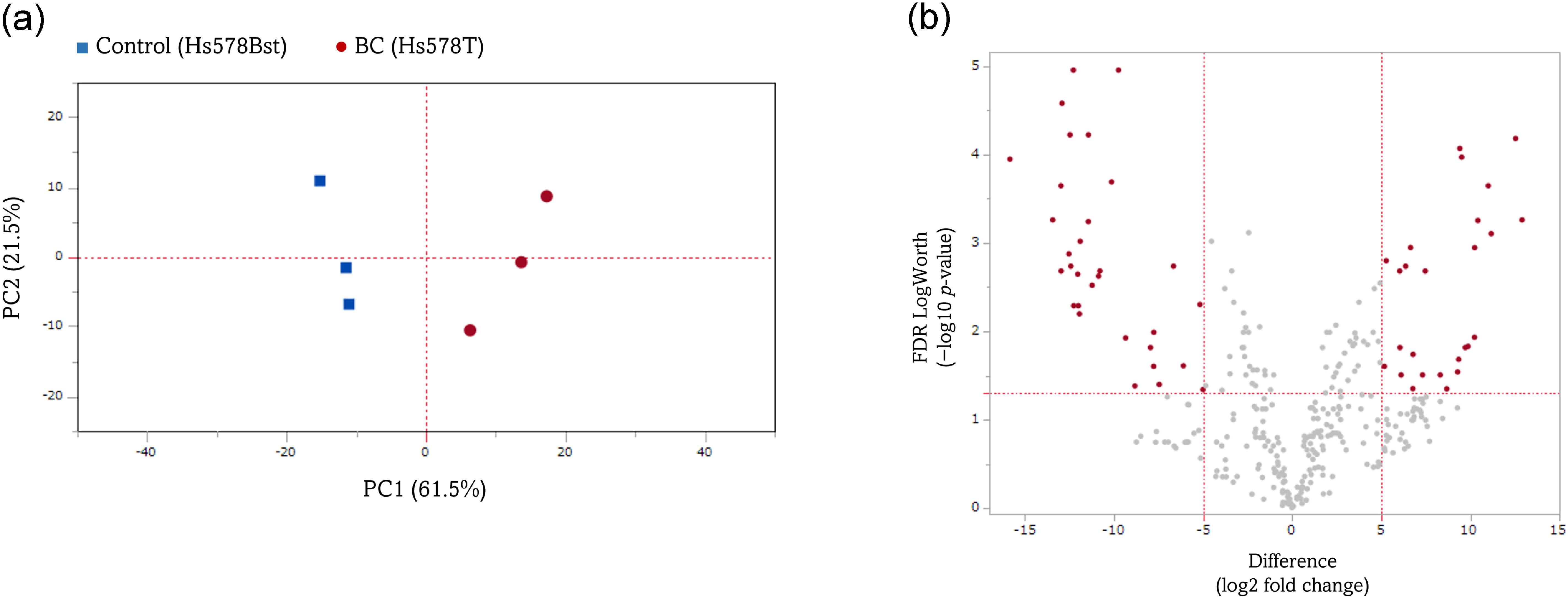
Fig. 3. Potential ability of the *N*-glycoproteins to distinguish between BC and control cell samples.

To confirm the ability of *N*-glycoproteins to distinguish between BC and normal cell lines, the PCA algorithm was implemented on the peak area values traced for the precursor ions of the *N*-glycoforms (Fig. 3a). Based on the scatter diagrams, BC and normal cells have separate data distributions. Accordingly, PCA revealed significant differences between the *N*-glycoform spectra of cell samples. Volcano plots revealed a comparative distribution of the peak area values traced for the precursor ions of the *N*-glycoforms in both cell samples (Fig. 3b). *N*-glycoforms with statistically significant differences (≥5-Difference, FDR Logworth ≥1.3) are indicated by red circles. Thus, in the 59 *N*-glycoforms from the 31 glycoproteins, the *N*-glycan moieties were changed in a region-specific manner. Some of these proteins may contain inconsistencies in *N*-glycosylation, such as lack of transmembrane helices or secretory signal sequences. Glycosylation validation using TMHMM and SignalP server further narrowed down these proteins, leading to 51 glycoforms derived from 27 glycoproteins for PPI network analysis (Table 1).

(a) Principal component analysis (PCA) of the peak area of the *N*-glycoforms. Three squares and circles represent each individual experiment. (b) Volcano plots of *N*-glycoforms in BC cell samples compared to those in control cell samples. Red dots: difference ≥5.00; FDR log Worth ≥1.3.

**Table 1:** Table 1. List of aberrant *N*-glycoforms in the BC and control cell samples.

Accession	Protein	Position	Glycan composition^a)^	Difference	FDR LogWorth
P15144	Aminopeptidase N	N128	7200	−12.3	2.3
		N234	5300	−7.8	1.6
P30530	Tyrosine-protein kinase receptor UFO	N198	5411	9.3	1.5
P08962	CD63 antigen	N130	8200	−10.1	3.7
Q96HE7	ERO1-like protein alpha	N280	7200	8.3	1.5
Q96AY3	Peptidyl-prolyl *cis*-trans isomerase FKBP10	N70	6200	−8.8	1.4
			5200	10.2	1.9
			3310	11.0	3.6
Q9Y680	Peptidyl-prolyl *cis*-trans isomerase FKBP7	N45	6200	6.6	3.0
P02751	Fibronectin	N542	9200	−5.2	2.3
O60565	Gremlin-1	N42	8200	−7.5	1.4
Q9Y4L1	Hypoxia up-regulated protein 1	N862	8200	6.0	1.8
			7200	7.3	1.5
		N869	8200	9.7	1.8
		N931	5200	9.3	1.7
P17936	Insulin-like growth factor-binding protein 3	N116	8200	−9.4	1.9
		N199	8200	−10.9	2.6
P08648	Integrin alpha-5	N868	8200	12.5	4.2
			7200	5.2	1.6
			5200	6.0	2.7
			5421	7.5	2.7
Q13683	Integrin alpha-7	N989	8200	−11.9	3.0
			7200	−12.3	5.0
			6200	−10.8	2.7
			5200	−12.5	4.2
			4200	−11.4	3.2
			3200	−6.7	2.7
P06756	Integrin alpha-V	N554	4610	6.1	1.5
P05556	Integrin beta-1	N520	9200	−11.4	4.2
P07942	Laminin subunit beta-1	N677	8200	9.4	4.1
			6200	9.5	4.0
P11047	Laminin subunit gamma-1	N1107	6200	6.4	2.7
		N1395	6200	5.3	2.8
			5200	12.9	3.3
			4200	11.2	3.1
P11279	Lysosome-associated membrane glycoprotein 1	N84	4311	−11.2	2.5
		N261	6300	−7.8	2.0
Q07954	Prolow-density lipoprotein receptor-related protein 1	N1825	7200	−9.8	5.0
Q5JRA6	Transport and Golgi organization protein 1 homolog	N246	6200	6.8	1.7
Q86UE4	Protein LYRIC	N175	6200	−6.1	1.6
P14314	Glucosidase 2 subunit beta	N72	6200	9.9	1.8
Q96D15	Reticulocalbin-3	N140	6200	−15.8	4.0
			5200	−13.0	2.7
			3210	−12.0	2.7
			3310	−12.4	2.7
P09486	SPARC	N116	5200	8.7	1.4
			4200	10.2	3.0
P02786	Transferrin receptor protein 1	N727	7200	6.8	1.4
P07996	Thrombospondin-1	N1067	5200	10.4	3.3
Q9BVK6	Transmembrane emp24 domain-containing protein 9	N125	5411	−11.9	2.2
O60637	Tetraspanin-3	N167	6300	−5.0	1.3

^a)^ *N*-glycan composition is presented based on the number of hexoses, *N*-acetylhexosamines, deoxy-hexose, *N*-acetylneuraminic acid.

### BC-related intracellular pathways

**Figure 4:**
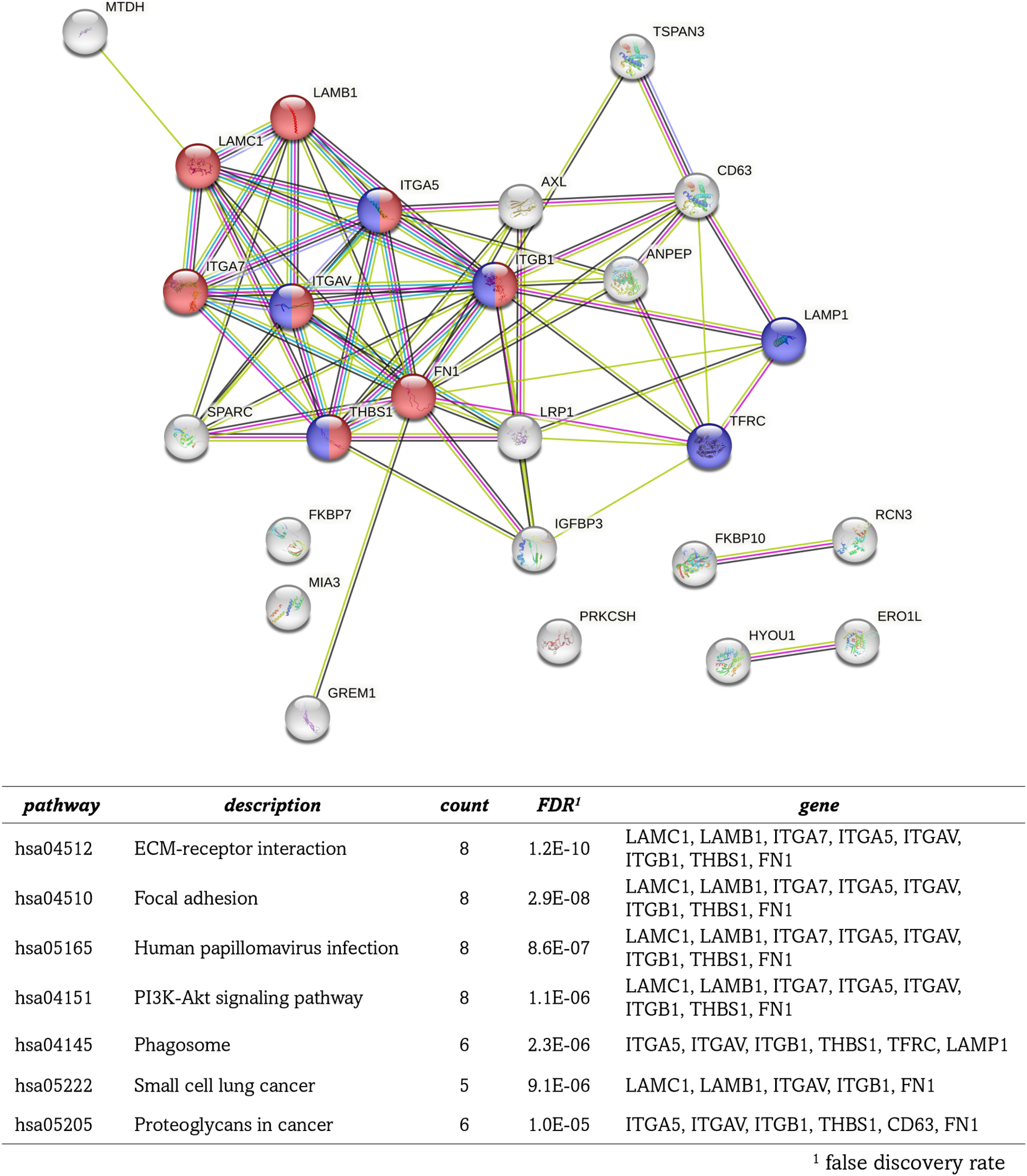
Fig. 4. Illustration of the PPI network of the 27 glycoproteins with site specific glycosylation changes.

Figure 4 shows the PPI network of the 27 glycoproteins with site specific glycosylation changes. The PPI network with clear interactions for these *N*-glycoproteins included 26 nodes and 68 edges. The top five pathways with the lowest FDR were ECM-receptor interaction (KEGG pathway: hsa04512, count=8, FDR=1.2E-10), focal adhesion (KEGG pathway: hsa04510, count=8, FDR=2.9E-8), human papillomavirus infection (KEGG pathway: hsa05165, count=8, FDR=8.6E-7), PI3K-Akt signaling pathway (KEGG pathway: hsa04151, count=8, FDR=1.1E-6), and phagosome (KEGG pathway: hsa04145, count=6, FDR=2.3E-6). It is noteworthy that eight common glycoproteins, laminin subunit beta-1 (LAMB1), laminin subunit gamma-1 (LAMC1), integrin alpha-5 (ITGA5), integrin alpha-7 (ITGA7), integrin alpha-V (ITGAV), integrin beta-1 (ITGB1), fibronectin (FN1), and thrombospondin-1 (THBS1), are commonly involved in the first four intracellular pathways. Additionally, ITGA5, ITGAV, ITGB1, THBS1, transferrin receptor protein 1 (TFRC), and LAMP1 are involved in phagosomes.

Proteins marked in red are associated with the PI3K-Akt signaling pathway, and those marked in blue are associated with the phagosome.

### Site-specific glycosylation changes in BC cells

**Figure 5:**
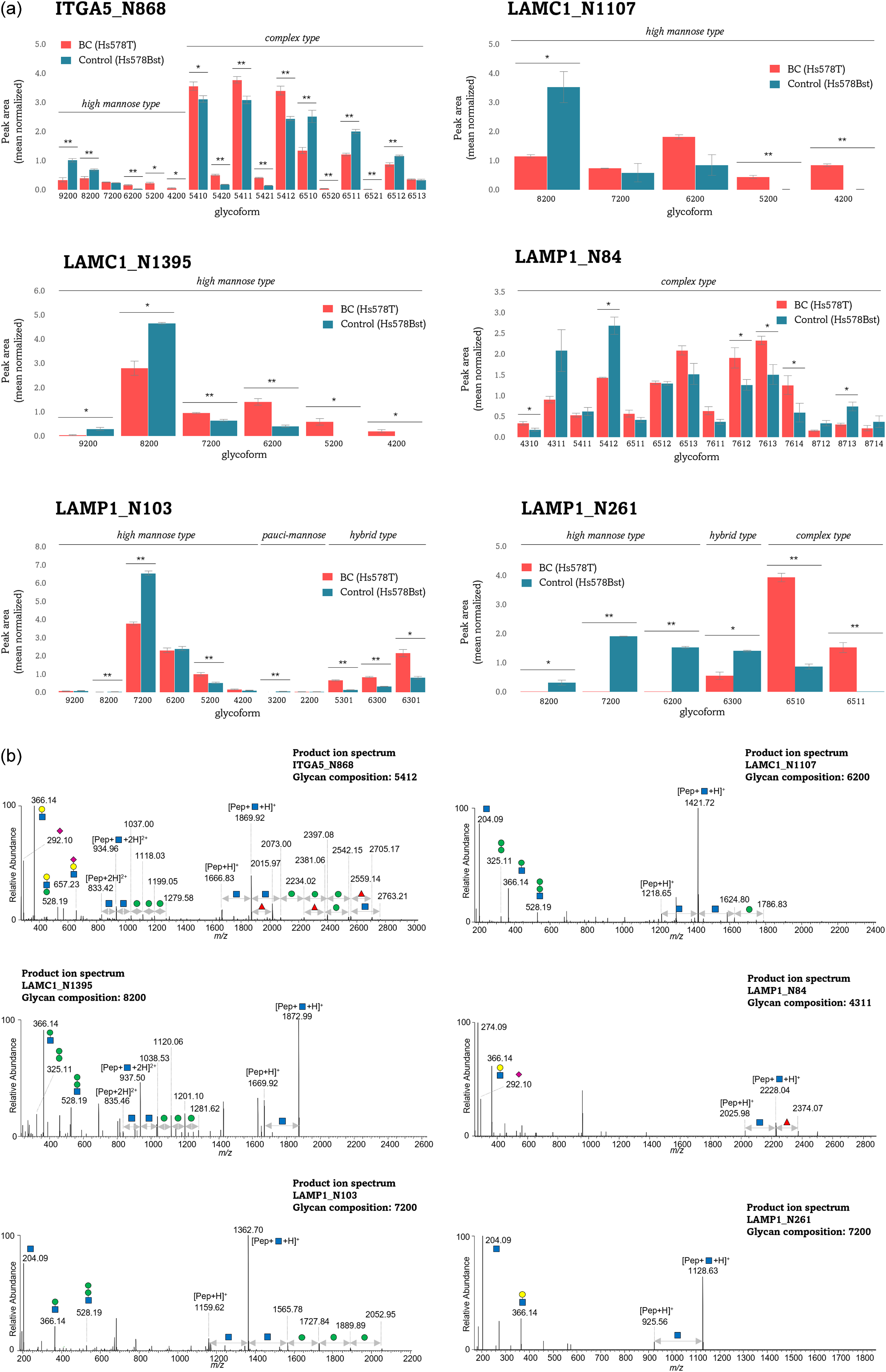
Fig. 5. Changes in site-specific glycosylation in BC cells.

*N*-Glycosylation changes occur mainly in proteins involved in limited intracellular pathways, such as the PI3K-Akt signaling pathway and phagosomes. To assess the site-specific *N*-glycosylation changes, we identified three aberrant *N*-glycoproteins, ITGA5, LAMC1, and LAMP1, involved in these intracellular pathways. Figure 5a shows a comparison of the mean normalized peak area values of site-specific glycan profiles between BC and control cell samples. Representative changes in *N*-glycosylation included the trimming of terminal mannose (N1107 and N1395 of LAMC), an increase in bi-antennary complex-type glycans (N868 of ITGA5), a decrease in the polylactosamine chain (N84 of LAMP1), and the conversion of high-mannose type glycans to hybrid-type glycans (N261 of LAMP1). Glycosylation of these remarkable proteins was supported by the product ion spectra (Fig. 5b). The typical oxonium ions are annotated on spectra including *m*/*z* at 204.08 (HexNAc), 292.10 (NeuAc), 325.11 (Hex+Hex), 366.14 (HexNAc+Hex), and 657.23 (HexNAc+Hex+NeuAc). The ions corresponding to a peptide ion carrying a single *N*-acetylglucosamine, and glycan-related ions that were correctly interpreted, have a high share of the total intensity in their spectra. The relative expression levels of the remarkable glycoproteins were analyzed by comparing the average peak areas ratios of the deamidated peptide ions from the two cell lines (Table 2). Based on the tumor-to-normal (T/N) peak area ratio of deamidated peptide ions, ITGA5, LAMC1 were upregulated in cancer cells with a T/N of over 2.0, whereas LAMP1 was downregulated in the cancer cells with a T/N ratio of less than 0.7 (Table 2). Using the LAMP1 as representative glycoprotein, the quantitative changes between the cell lines analyzed by LC/MS/MS were supported by western blot analysis (Supplementary Figure S1). LAMP1, which shows a remarkable change in *N*-glycosylation, was selected and subcellular localization analysis was performed.

(a) Quantitative comparison of *N*-glycosites. The *N*-glycan composition was determined based on the number of hexoses, *N*-acetylhexosamines, deoxy-hexose, *N*-acetylneuraminic acid. (b) Product ion spectra of the *N*-glycopeptides. Symbols: green circle, mannose; blue square, *N*-acetylglucosamine; red triangle, fucose; yellow circle, galactose; purple diamond, *N*-acetylneuraminic acid.

**Table 2:** Table 2. Relative expression levels of the remarkable glycoproteins.

Protein name (Gene symbol)	Sequence	Glycosite	Ratio^a)^
Integrin alpha-5 (ITGA5)	VTGL*N*CTTNHPINPK	N868	3.9
Laminin subunit gamma-1 (LAMC1)	V*N*NTLSSQISR	N1107	2.7
	KIPAI*N*QTITEANEK	N1395	2.1
Lysosome-associated membrane glycoprotein 1 (LAMP1)	E*N*TSDPSLVIAFGR	N84	0.7
	GHTLTL*N*FTR	N103	0.4
	LLNINP*N*K	N261	0.4

^a)^ Peak area ratio of de-glycosylated peptides in tumor *vs.* normal cells (T/N ratio). *N*, glycosylation site on the sequence.

### Subcellular localization of LAMP1 in BC cells

**Figure 6:**
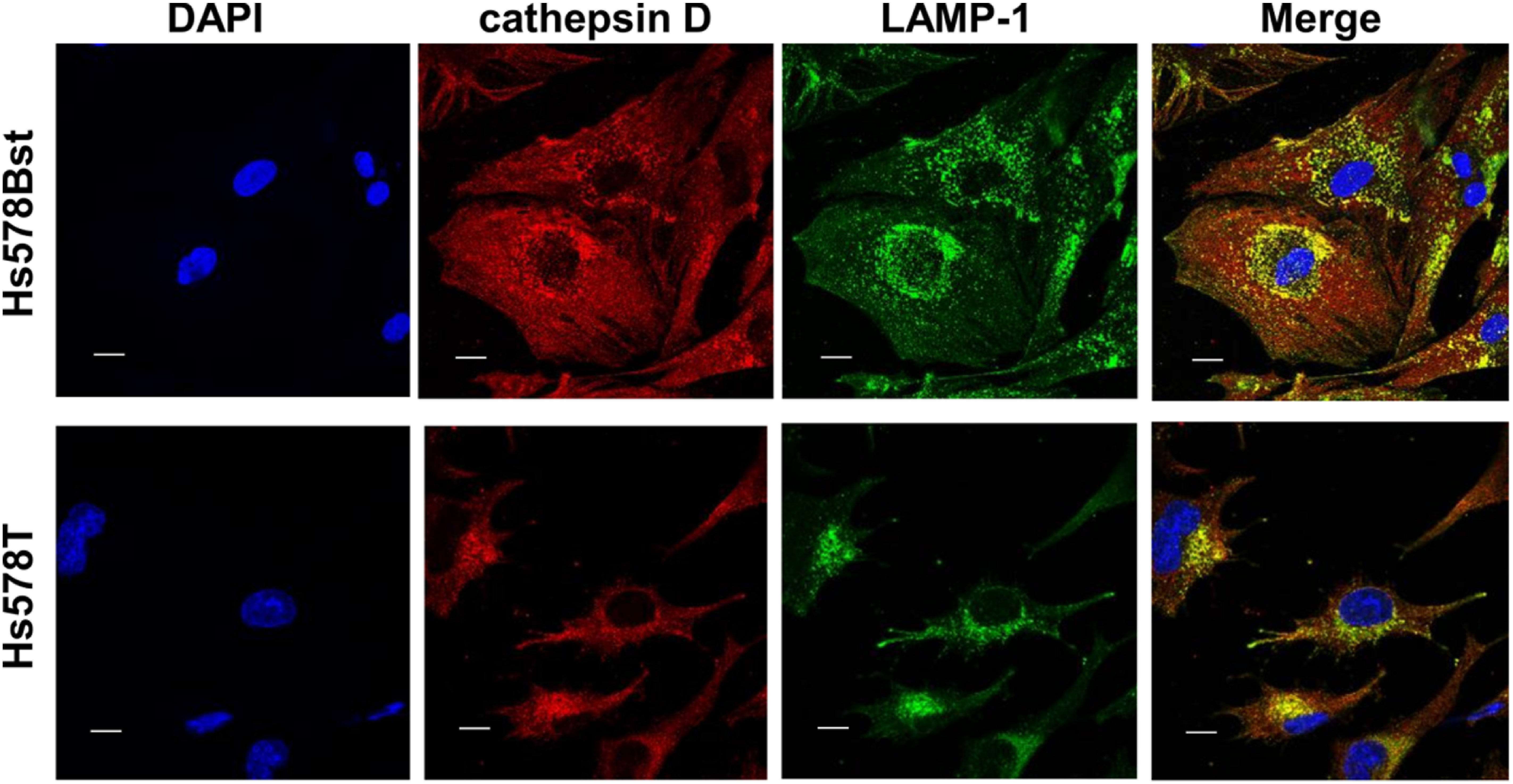
Fig. 6. Subcellular localization analysis of LAMP1 based on immunofluorescence staining in BC and control cells.

LAMP1 is a major protein in the lysosomal membrane and is expressed in the plasma membranes of malignant cells. To determine the influence of dynamic changes in glycosylation, we examined the subcellular localization of LAMP1 in BC and control cells (Fig. 6). Confocal immunofluorescence of cathepsin D, which is used as a lysosome marker, revealed that lysosomes were distributed throughout the cells in the control group, but had accumulated in the perinuclear space in cells in the BC group. Co-localization of LAMP1 and cathepsin D was observed in both cell lines.

Both cell types were simultaneously stained with cathepsin D (red) and LAMP1 (green) monoclonal antibodies. The nuclei were stained with 4′,6-diamidino-2-phenylindole (blue). Scale bars=17 μm.

## DISCUSSION

In this study, we conducted *N*-glycoproteomics in an attempt to elucidate the relationship between glycosylation changes and malignant transformation of BC cells. Glycopeptides were enriched from the membrane protein fractions of representative BC cell lines and their syngeneic cell lines using an acetone-based glycopeptide enrichment method. All of the *N*-glycoforms that were identified by the Byonic search engine were compared between the BC and control cells. As shown in Fig. 3, PCA using the peak area values of the identified *N*-glycoforms revealed separate clusters in BC and control cells. The differences in *N*-glycoforms between the two cell types suggest that cancer-specific glycan alterations are due to the aberrant substrate selection of various glycosyltransferases in BC cells. In this study, the *N*-glycoforms of 27 proteins were found to be significantly altered in the BC cell line. In addition, these 27 abnormal *N*-glycoproteins were found to be associated with the PI3K-Akt pathway and phagosomes. These intracellular pathways are not independent of BC. PI3K-Akt is a major signaling pathway that is involved in regulating cell proliferation, survival, and metabolism, but is often the most activated oncogenic pathway in triple-negative breast cancer (TNBC).^29–31)^ Therefore, clinical trials based on PI3K-Akt inhibition are currently underway.^32)^ Further, the epithelial–mesenchymal transition of TNBC cells *via* the PI3K/Akt pathway involves the *N*-glycosylation of cell surface proteins.^33)^ In contrast, autophagy is maintained at high levels in TNBC cells, and the knockdown of relevant genes significantly suppresses cell proliferation, colony formation, migration/invasion, and induces apoptosis of TNBC cells.^34,35)^ Cell cultures under different conditions may affect protein glycosylation. However, it should also be noted that the changes in glycosylation are found in glycoproteins, which seem to be primarily related to the PI3K-Akt pathway and phagosomes.

As shown in Fig. 5a, the *N*-glycan profiles of LAMP1 had the most remarkable change in BC cells. Tetra-antennary *N*-glycans with a polylactosamine structure were found on Asn84 in LAMP1: Hex_8_HexNAc_7_dHex_1_NeuAc_2–4_. Our data are consistent with findings reported in previous studies that showed that LAMP1 is one of the most important 1,6-branched polylactosamine-carrier proteins in chronic myelogenous leukemia cells.^36)^ A decrease in the polylactosamine structure, which protects LAMP1 from the action of lysosomal hydrolases, in BC cells may result in the degradation of LAMP1 and the concomitant destabilization of lysosomal function.^37)^ As shown in Fig. 6, lysosomes containing LAMP1 predominantly accumulated in the cytoplasm around the nucleus. The spatial distribution of lysosomes is involved in cancer cell metastasis and drug resistance.^38,39)^ These irregular lysosomal distributions may not be independent of the remarkable changes in LAMP1 glycosylation.

In conclusion, we employed *N*-glycoproteomics to demonstrate the aberrant *N*-glycosylation in BC cell samples and to identify the intracellular pathways affected by this process. Membrane protein *N*-glycosylation was found to clearly differ between BC and control cell samples, and aberrant *N*-glycosylation at specific sites may contribute to cell proliferation and survival. Further, the diversity of site-specific glycan changes suggests perturbation of glycosyltransferase substrate recognition in BC. Further validation using clinical tissue specimens and detailed site-specific *N*-glycosylation analysis may enable the development of new therapeutics for BC.

## Notes

Mass Spectrom (Tokyo) 2023; 12(1): A0117
