# Supplementary figures and images for: Comprehensive Membrane N-Glycoproteomics Using Human Breast Cancer Cell Line Pairs
Source: Mass Spectrom (Tokyo). 2023 Apr 11;12(1):A0117. doi: 10.5702/massspectrometry.A0117 (PMC10213999; doi:10.5702/massspectrometry.A0117)

***Control***   ***BC***

---

130—

95—

kDa

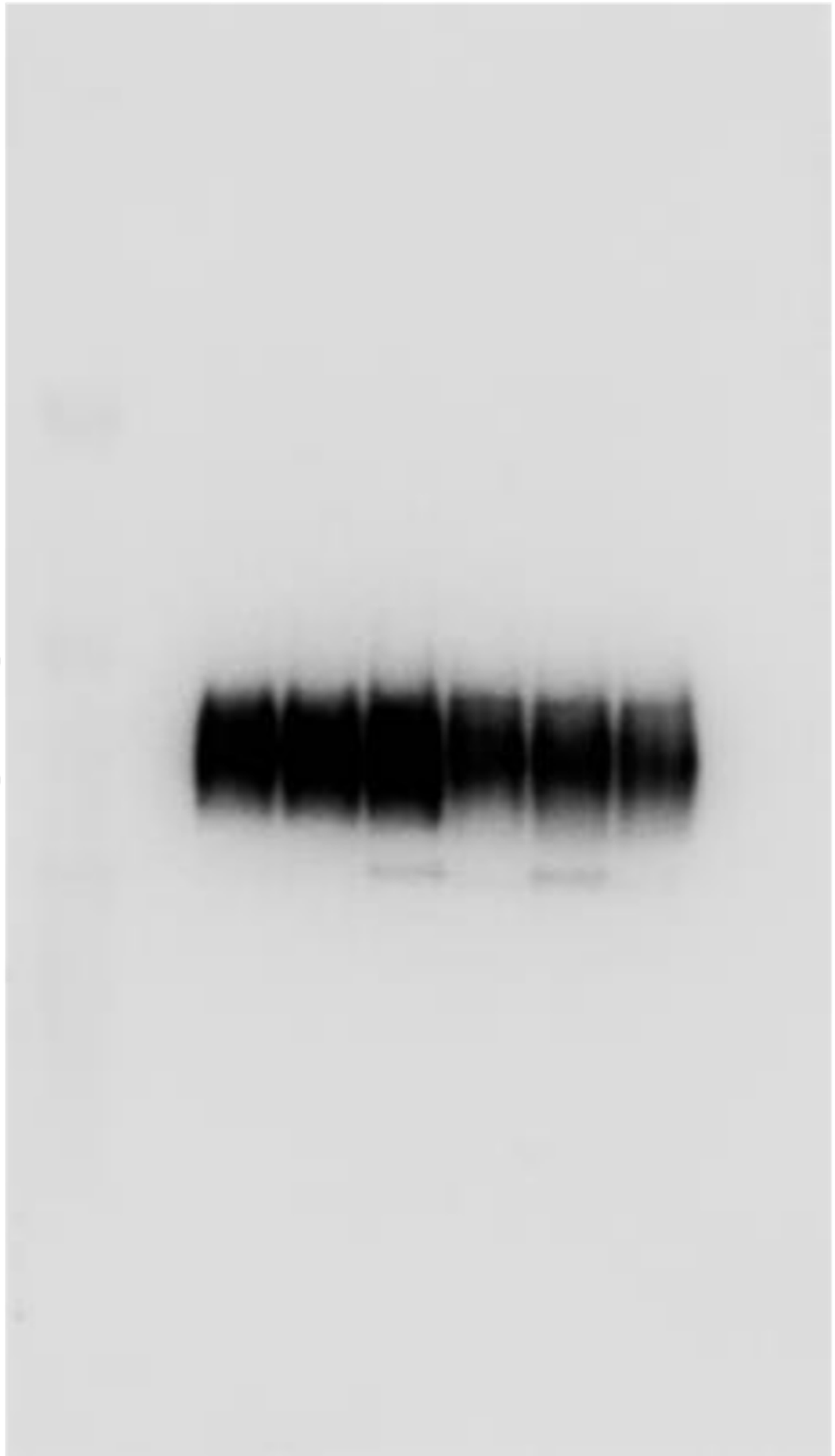

**WB: anti-LAMP1**

Supplement: Supplementary Data [file massspectrometry-12-1-A0117_s001.pdf]
